# A Novel Insect Overwintering Strategy: The Case of Mealybugs

**DOI:** 10.3390/insects14050481

**Published:** 2023-05-19

**Authors:** Rosa Vercher, Sandra González, Adrián Sánchez-Domingo, Juan Sorribas

**Affiliations:** 1 Escuela Técnica Superior de Ingeniería Agronómica y del Medio Natural, Universitat Politècnica de València, Camino de Vera, s/n, 46022 Valencia, Spain; rvercher@eaf.upv.es (R.V.); sangonc1@upvnet.upv.es (S.G.); adsando1@alumni.upv.es (A.S.-D.); 2 Universitat Politècnica de València, Camino de Vera, s/n, 46022 Valencia, Spain

**Keywords:** insect behaviour, cold avoidance, belowground insect, ground overwintering, insect sexual dimorphism, root-feeding insect, wintering, mealybugs biology, *Planococcus citri*, *Pseudococcus viburni*

## Abstract

**Simple Summary:**

The mealybugs are a large family of sap-sucking herbivore insects comprising more than 2000 species. They feed on many economically important agricultural and ornamental plants. During the cold periods of the winter, insects overwintering without migrating often require special habitats to survive unfavorable environmental conditions. In the case of mealybugs inhabiting fruit orchards, it was believed that the trunk and the branches were the main overwintering sites where individuals diapause. Here it is shown for the first time that, in temperate climates, mealybugs spend half of the year on the tree canopy and the large majority move to the rhizosphere of the fruit tree during the winter to feed on the roots and reproduce (complete at least one generation) under the new “belowground habitat.” This unique adaptive strategy allows an aboveground herbivore to turn into a belowground root-feeding insect for part of the year. The preferred area to overwinter for the main female mealybug species is within 1 m in diameter around the fruit tree trunk, where more than 12,000 mealybug males per square meter can emerge every spring. These findings have implications at the ecological and agronomical levels since current treatments to control mealybug pests are based on the fruit trees’ canopy.

**Abstract:**

Insects have limited ability to regulate their body temperature and have thus required a range of strategies to withstand thermally stressful environments. Under unfavorable winter conditions, insects often take refuge under the soil surface to survive. Here, the mealybug insect family was selected for the study. Field experiments were performed in fruit orchards in eastern Spain. We used specifically designed floor sampling methods combined with fruit tree canopy pheromone traps. We found that in temperate climates, the large majority of the mealybugs move from the tree canopy to the roots during the winter, where they turn into belowground root-feeding herbivores to survive and continue underground the reproductive process. Within the rhizosphere, mealybugs complete at least one generation before emerging on the soil surface. The preferred area to overwinter is within 1 m in diameter around the fruit tree trunk, where more than 12,000 mealybug flying males per square meter can emerge every spring. This overwintering pattern has not previously been reported for any other group of insects showing cold avoidance behavior. These findings have implications at the winter ecology level but also at the agronomical level since treatments to control mealybug pests are, until now, only based on the fruit trees’ canopy.

## 1. Introduction

Insects, as cold-blooded ectothermic animals, have limited ability to regulate their body temperature, which depends largely on ambient temperature, so insects use behavior to adjust their temperature to some degree [1]. In temperate zones, temperature drops during the winter months trigger different responses in insects to survive the cold. Insects overwintering without migrating often require special habitats to survive unfavorable environmental conditions [2].

The insect winter ecology has traditionally been divided into two main groups and several overwintering strategies that can be observed: “freeze tolerant” and “freeze avoiding.” Some overwintering insects replace the water in their bodies with glycerol or polyhydroxyl alcohols, which act as antifreeze to make them freeze tolerant [3], while many other insects partially avoid the cold by hiding in protected, warmer locations such as leaf litter, the bark of certain trees or under the soil surface (e.g., [4]). The group of insects showing the ability to overwinter in the ground can be further split into two subgroups: (1) insects that have evolved to enter the diapause phase (usually pupal diapause) as soon as they penetrate into the ground and find a suitable place and (2) insects that can feed within the rhizosphere before or during the overwintering phase.

Many examples can be found for the first group of insects, such as the medfly, *Ceratitis capitata,* whose larvae, upon arrival at ground level, transform immediately to pupal stage and remain in diapause until the climate is suitable for adult emergence [5]. Further examples are the potato beetle, *Leptinotarsa decemlineata* [6] or the tobacco budworm, *Heliothis virescens* [7].

Examples of the aforementioned second group are more scarce (or less known). The lupine ghost moth, *Hepialus californicus,* whose larvae develop and feed on the lupine root, has one generation per year. Pupation occurs in winter, and pupal diapause lasts until the adult emergence in the spring [8]. Another species, the grape root borer, *Vitacea polistiformis*, feeds on the roots of grape vines. The life cycle takes 2 years to complete, and almost all of it is spent as larvae. Flying adults emerge at the beginning of the summer after an obligate pupal diapause [9]. A third example is the cabbage root fly, *Delia radicum*, whose larvae feed on the roots and stems of cabbage plants. After overwintering as pupae in the soil, adult flies emerge in the spring [10].

Species of the last mentioned group are obligated insect root herbivores, and their flying adults emerge for mating after a pupal diapause phase. However, there is a particular family of insects showing a different pattern of behavior than any other, the mealybugs (Hemiptera: Pseudococcidae). Females of several mealybug species have been reported to migrate during the winter from the plant canopy toward the basal parts of the trunks, mostly on citrus trees and vineyards [11,12,13,14]. However, until now, the presence of mealybugs at ground level was considered accidental and insignificant, and studies stated that the tree canopy and/or the trunk are the main overwintering areas [15,16,17,18].

Mealybugs are sap-sucking insects feeding on leaves, stems and twigs of many species of shrubs, trees and grasses [19]. They excrete large quantities of honeydew that encrust the leaves and clusters, resulting in further crop damage and defoliation. Molds colonize the honeydew-coated leaves causing them to look dark, reducing fruit quality and lowering tree vitality through the loss of photosynthetic capacity [20]. Many mealybug species are economically important agricultural and ornamental pests feeding on plants such as citrus, sugarcane, pineapple, papaya, cotton, pear, mulberry, sunflower, cacti, gardenias and orchids [19,21,22,23]. Mealybugs are widespread in Africa, where the casava and the papaya mealybugs have been reported as serious economic pests [24,25]. Many invasive mealybugs have been cited as pests of agriculture crops in temperate and subtropical areas of North America and South America (for instance [26,27]). In Asia, the cotton mealybug, *Phenacoccus solenopsis,* is found in many temperate areas and many crops, and many other mealybugs affecting agricultural crops have been cited [23]. All over the Mediterranean basin, mealybugs have been reported as a key pest of many crops; nevertheless, mealybugs feeding on subtropical fruit trees (mainly citrus, mango, papaya and persimmon trees) and vineyards are the most widely studied due to their wide distribution and severe pest effects on these plants [11,14,15,17,28].

The occasional finding of mealybugs on the soil surface or underground has been associated with fruit tree dropping [15,29]. Studies trying to find overwintering mealybugs belowground did not obtain any results in this respect [14,16,17,18]. Accordingly, female progeny have been described to migrate during the spring from the trunk and bottom parts of the plants towards the fruit tree canopy [11,12,14,29]. Thus authors assumed that ground presence was accidental and irrelevant.

In this study, mealybugs were chosen as a model for a new group of insects with a previously undescribed behavior: insects that spend half of their lifecycle feeding on the aerial parts of the plant and change their main developing environment for that of the subterranean rhizosphere environment to overwinter while continuing to feed and develop. We conducted specifically designed field experiments in order to evaluate if the rhizosphere represents an essential environmental medium for mealybugs during the winter months, during which they continue their reproductive process. Besides, we aimed to know how mealybugs move from the basal parts of the trees toward the fruits throughout the year.

## 2. Materials and Methods

### 2.1. Studied Organisms

Mealybugs are a large family of *Coccoidea* insects comprising more than 2000 species worldwide distributed [21]. They are sexually dimorphic: the adult female mealybug is wingless and is covered with a white-cottony wax. However, male mealybugs are smaller and do exhibit a radical change during their life cycle, changing from wingless, ovoid nymphs to wasp-like flying adults [21,22].

As a model for this study, we used the binome mealybugs-persimmon fruit trees. The oriental persimmon tree, *Diospyros kaki*, native of China, has become widespread in the Mediterranean basin during the last decades [28,30,31] and in many countries such as the Republic of Korea, Japan, Brazil, Chile, the United States, and Australia [18,31,32,33,34]. In most of these countries, mealybugs have been reported as a serious pest, and this is also the case in eastern Spain [35,36], the region where this study was performed. In Spain, the citrus mealybug *Planococcus citri* and the obscure mealybug *Pseudococcus viburni* have been cited as the most abundant mealybugs in persimmon orchards [37,38], however recent studies have shown that in some areas these species have been outcompeted by the newly arrived *Pseudococcus longispinus,* the long-tailed mealybug [39] and *Delottococcus aberiae* [40]. *P. citri*, the most widely studied species, can complete 5 generations per year in Spanish citrus/persimmon growing areas, and its lower developmental threshold temperature is 8.3 °C [41].

### 2.2. Study Area and Sampling Stages

Field studies were performed in the Valencia region, a traditional Spanish agricultural area located in the western Mediterranean basin. The landscape of the study area is composed mainly of small citrus and persimmon orchards of <1 ha spread over an extension of about 160,000 ha in an almost continuous belt, 300 km long from north to south and about 60 km wide. The area has a typical Mediterranean coastal climate with temperatures seldom below 0 °C during winter due to the influence of the sea.

The persimmon production area has been increasing since 1998, up to about 18,000 ha in 2019, replacing, in many cases, the previous citrus orchards [30,42]. In Spanish citrus groves, mealybugs have been considered one of the main pests for many years, especially the citrus mealybug *P. citri* [43,44]. In the Valencia region, mealybugs are so common and widely distributed in fruit trees and ornamental plants that they have a local name, namely “cotonet,” different from the Spanish names “cochinilla algodonosa” or “chanchito blanco.”

**Figure 1:**
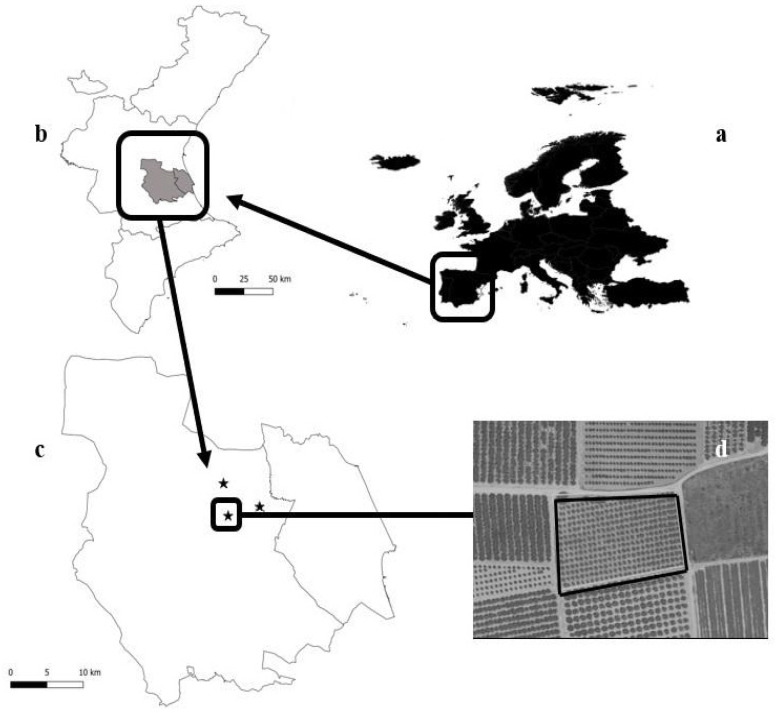
Maps showing the geographic location of the fruit orchards used for field experiments: (**a**) the Valencia region in eastern Spain. (**b**) Ribera County, the main area for commercial persimmon orchards in Valencia. (**c**) Sites of experimental orchards are indicated with stars. (**d**) Satellite imagery of the selected persimmon orchard for below-ground experiments.

From 2018 to 2020, we conducted 3 consecutive field experiments. The survey was scheduled in progressive form, and the results of the first experiment during the first year were the basis for the design of the sampling protocol for the second experiment that started in 2019. Similarly, the results of the second experiment were the basis for the sampling protocol for the third experiment. Initially, we sampled 3 persimmon orchards belonging to 3 villages, namely Alginet, Sollana and Algemesí, all about 20 km apart (see Figure 1). Orchards were similar in size, between 0.5 and 0.7 ha, which is the typical orchard size in this region. This was the prior trial before the start of the so-called “belowground experiments” in order to have a picture of the mealybug species composition in the area of study, their relative abundance and population dynamics. In the second year, we selected one orchard for the specifically designed ground sampling and in the third year, we adapted the methodology and dates for the sampling of insect rhizosphere distribution. Therefore the sampling process was divided into 3 stages: (1) Sampling for species identification and abundance from 2018 to 2020. (2) Sampling for evaluating below-ground mealybugs from 2019 to 2020. (3) Sampling to determine mealybugs distribution in the rhizosphere during 2020. All orchards were cultivated under integrated pest management according to EU rules, and no treatments against mealybugs were performed. Orchards had been under identical farming practices for at least the previous 5 years.

### 2.3. Tree Canopy Mealybugs

Two mealybug sampling methods were used on the canopy of persimmon trees: (1) delta traps for males and (2) direct visual observation of fruits for females.

Delta traps consist of a plastic trap folded into a triangle with a suspension hook hung on a branch and with a sticky insert where female sex pheromones are placed. Five traps, baited with *P. citri* (OpenNatur, Pherobank^®^, Lleida, Spain) and five with *P. viburni* female sex pheromones (OpenNatur, Pherobank^®^) were placed in the canopy of 10 trees randomly selected (field boundaries were avoided) in every targeted orchard. These pheromones were selected on the basis of previous results of other studies in fruit orchards of the Valencia region [38]. The traps were collected and replaced every week from May to November and once per month during the coldest period for this region, i.e., December to April, for 3 years. The trap sampling during the second and third years was intended to compare captures and seasonal population dynamics with those obtained from belowground sampling during the same period. A total of 1048 delta traps were collected over 3 years. In the laboratory, traps were examined and captured mealybug males were identified to species level and counted.

Fruits visual inspection of calyx and fruit surface was performed from the fruit set (phenology 77, developing fruit according to the BBCH scale) up to the end of the sampling period or until fruit harvest if that was the case. Fruits of citrus and persimmon trees are described to attract and concentrate mealybugs [17,41,45]. For each sampling day and orchard, 100 persimmon fruits were randomly selected from 10 trees (that is, 3000 fruits were visually examined per day unless fruit harvesting had started). When present, mealybugs were annotated, and adult females were identified to the species level. The taxonomic identification of the mealybug species was based on the morphological characteristics of the adult female [46,47]. We were advised on mealybugs identification by two expert colleagues, authors of a Pseudococcidae taxonomic group guidebook [48]. The fruit observation was performed on the same day on which traps were replaced (if fruits were available). Adult females on the fruits were identified to species level and counted.

Taking into account the sharp decline in the abundance of mealybugs on fruits and traps at the beginning of the winter and the low level of mealybugs observed on the leaves, branches or tree trunks, we assumed that at least some of them were overwintering in the rhizosphere. This fostered the idea of developing sampling methods to evaluate the presence of mealybugs belowground. We use the results of these pre-assays to determine the sampling period and the best pheromone for capturing mealybugs overwintering in the ground.

### 2.4. Belowground Mealybugs

For this experiment, we selected the orchard with the highest levels of mealybugs during the first year of canopy sampling, located in Sollana. During the autumn and winter of 2018, we dug a total of 30 holes, roughly 0.2 × 0.2 m in size, at distances varying from the tree trunk of the examined trees until we found the roots and gently removed the soil around them. We only observed 5 female mealybugs in some of the roots, which confirmed their ground-level presence (as observed by other authors), and 2 more attached to the roots of a *Parietaria* spp. spontaneous plant. However, this method is very costly, laborious and difficult to standardize since we don’t know previously where the roots are located in the ground. In addition, some mealybugs can be accidentally removed with the soil. Similar problems were described by Bignell et al. 2018 [18] using this mealybug sampling method.

We then tested a new sampling method specifically designed for detecting soil-emerging mealybug males. In light of the data obtained from the previous experiment, the sampling period started in June 2019 and ended in October 2020. We used hard plastic trays (50 × 30 cm and 10 cm), which were placed in an upside-down position on the soil surface after removing leaf litter or any grass presence. At the top of the inside part of the trays, we attached a 10 × 25 cm sticky plastic trap baited with *P. citri* pheromones. This pheromone was selected, taking into account the results of the previous experiment showing that the vast majority of the mealybugs in this area are *P. citri*. Trays were then placed 25 cm away from the persimmon tree trunk. To avoid the lateral entrance of any insects, the lateral borders of the trays were covered and sealed with loose soil. The experiment was replicated in 2 trees for the period 2019–2020 and in 4 trees in 2020, all of them spaced about 30 m apart. Since ants can tend and/or attack mealybugs [49,50,51] and thus interfere with the experiment, we also checked that there were no ants present near the trays. Traps were collected and replaced weekly from May to November and at least once per month during the rest of the year. In the laboratory, all traps were examined, and mealybugs were identified and counted.

### 2.5. Mealybug Root Preference

We used the results of the previous experiment confirming the belowground abundance of mealybugs and the period of male emergence from the ground to design an experiment to evaluate the preferred root area (or root size) by mealybugs to feed and overwinter.

**Figure 2:**
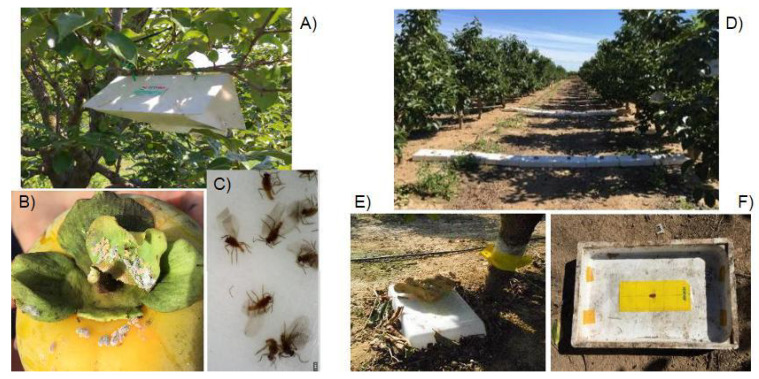
Schematic figure showing the sampling methods used to evaluate mealybug species composition and their seasonal evolution in the area of study (**left side**) and the specifically designed sampling methods to evaluate the presence of mealybugs belowground (**right side**). From top to bottom: (**A**) Delta pheromone trap on a persimmon tree, (**B**) female mealybugs on a persimmon fruit and (**C**) mealybug males captured on a trap; (**D**) two lines of trap trays joining persimmon trees of two contiguous field rows, (**E**) one individual trap tray placed next to the tree trunk and a (**F**) pheromone trap attached to the top of the inside part.

From June to August 2020, we placed on the ground 8 plastic trays side-by-side, forming a continuous line that filled the gap between two opposite trees of two contiguous tree rows. Trays were equal to that described in the previous experiment. The experiment was replicated twice with 10 m between tray lines (Figure 2). Traps were collected and replaced every week. We also checked there were no ants surrounding the trays. In the laboratory, all traps were examined, and male mealybugs were identified and counted.

### 2.6. Data Analysis

To standardize the results, the number of insects captured in all traps (at the tree canopy and ground levels) was divided by the number of days that each particular trap was in the field to obtain the average number of mealybugs captured per day.

The number of insects captured in the trap trays was correlated with the ground surface covered by one tray (0.15 m^2^) to obtain the estimated average number of mealybugs emerging per square meter.

All data were submitted for ANOVA (Analysis of Variance) at *p* = 0.05 level, followed by a Multiple Range Test for statistical analysis. Comparisons between the means were made using Fisher’s Least Significant Difference (LSD). The differences between the years and between plots (orchards) were analyzed in the same way. Data were log-transformed when necessary before ANOVA to stabilize the variance. Statistical analyses were performed using Statgraphics^®^ software (Statgraphics Centurion XVI).

## 3. Results

### 3.1. Tree Canopy Mealybugs

The total number of males captured in the 1048 delta traps collected over 3 years was 46,965 for *P. citri* compared to 2869 for *P. viburni*, meaning that more than 94% of the total mealybug males captured in the pheromone traps were *P. citri*.

**Figure 3:**
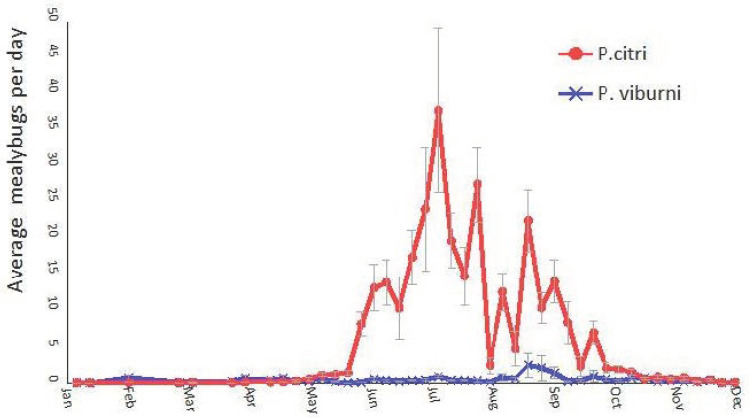
Seasonal dynamics at tree canopy level of the two main mealybug species, *Planococcus citri* and *Pseudococcus viburni* found on 3 commercial persimmon orchards of eastern Spain. The graphic shows the average number of male insects captured per day on Delta pheromone traps (see materials and methods) placed on branches of persimmon trees during the period 2019–2020. A total of 1048 delta traps were collected over 3 years. The total number of males captured were 46,965 *P. citri* and 2869 *P. viburni*. Vertical bars indicate standard error (SE) of the means.

Over the 3 years, the captures of *P. citri* mealybugs in traps started at the end of May and reached their highest peak in July before decreasing from September to almost nil during autumn and winter. The seasonal population dynamic of *P. viburni* was very different, with captures distributed all along the year, a peak of captures observed in September and no clear overwintering period. In order to compare with the results of the second experiment, we analyzed the trap captures separately for the period 2019–2020 (Figure 3).

A total of 28,400 fruits were examined. Based on adult females observed, three mealybug species were identified on persimmon fruits: *Planococcus citri*, *Pseudococcus viburni* and *Pseudococcus longispinus*. The relative abundance of mealybugs feeding on persimmon fruits (female stages) was: 98% *P. citri*, 2% *P. viburni*, and <0.1% *P. longispinus*. There were no statistical differences between the years (F_2,1045_ = 0.10; *p* = 0.90) or between plots (orchards) (F_2,1045_ = 1.20; *p* = 0.30). Within the studied orchards, *P. citri* and *P. viburni* coexisted in most trees (76%), while *P. longispinus* was only found in one of the studied orchards.

**Figure 4:**
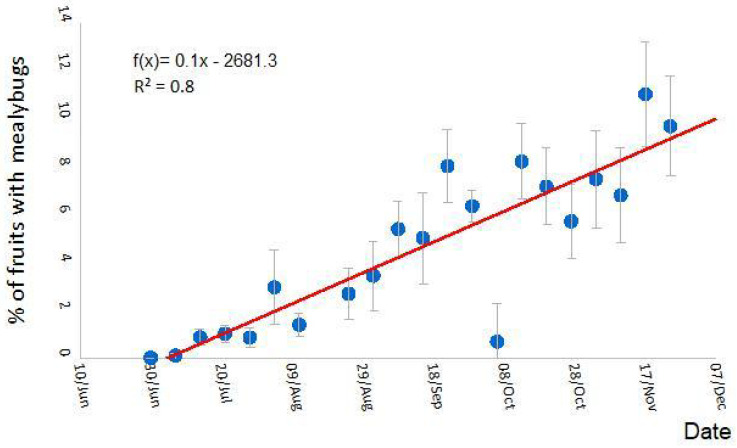
Seasonal trend of the percentage of persimmon fruits with the presence of *P. citri* female mealybugs (young mealybug females were assumed to be *P. citri* species, see results section). Fruit direct field examination was performed weekly. The sampling period started in May (although no mealybugs presence was detected until the beginning of July) and ended when fruits were harvested. Each point represents the average of 3 years, from 2018 to 2020, and three persimmon orchards in which 3000 fruits were visually observed per day and orchard and year. Vertical bars indicate the standard error (SE) of the means.

The presence of adult females on the fruits steadily increased from the beginning of July until the end of November. The percentage of fruits in which one or more mealybugs were observed is shown in Figure 4. Young females were assumed to be *P. citri* when direct field identification to species level was not possible. In most years, the fruit harvest was performed at the end of November or at the beginning of December; therefore, data collected in December were excluded from Figure 4.

### 3.2. Mealybug Belowground Survival

During the two years, 2019 and 2020, a total of 22,930 mealybug males were captured from the sticky traps positioned inside the trays placed 25 cm away from the persimmon tree trunk. Considering the size of the trays (the surface covered by one tray is 0.15 m^2^) and the total number of replicates (six), this means that per square meter, an estimated average of 12,740 ± 762 mealybug flying males can emerge every year (or every spring in other words).

**Figure 5:**
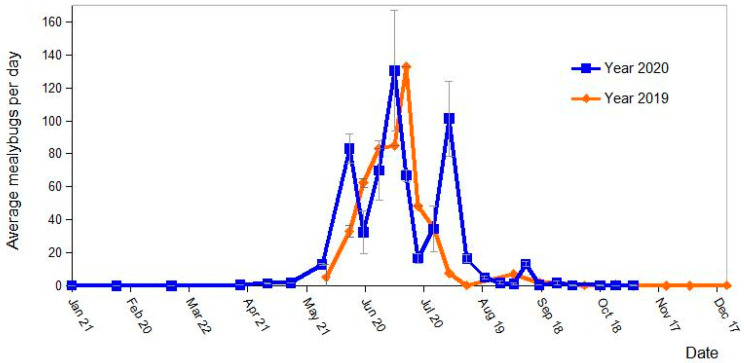
Average captures per day of Planococcus citri ground emerging males during the years 2019 and 2020. The sampling method was specifically designed using pheromone trap trays (see materials and methods) to evaluate the new generation of mealybugs emerging from the rhizosphere of 4 persimmon trees located in an orchard in eastern Spain. Vertical bars indicate the standard error (SE) of the means.

Captures reached the highest peak in July and decreased from August onwards to almost zero during the autumn and winter (Figure 5). Consequently, we stopped the sampling in October 2020. As expected, seasonal dynamics were fairly similar to those shown by the captures with Delta pheromone traps placed in the tree canopy. Curiously, for the trap trays in which the experiment took 2 years, the capture of mealybugs during the second year did not decrease, i.e., 7156 in 2019 and 8913 in 2020, considering the same time period. There were no statistical differences between captures in trays in any of the years when we considered the same time period (F_3,72_ = 0.18; *p* = 0.91).

### 3.3. Mealybug root Preference

**Figure 6:**
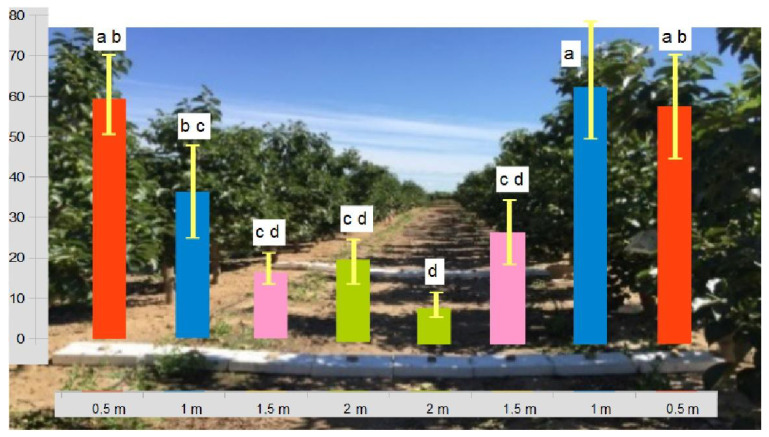
Average captures per day of Planococcus citri ground emerging males during the year 2020 at several distances from two persimmon trees. The sampling method was specifically designed (see materials and methods) to evaluate the new generation of mealybugs emerging from the rhizosphere. We used eight pheromone trap trays forming a continuous line from two opposite trees of two contiguous field rows. Traps were placed at 0.5, 1, 1.5, and 2 m (the center of the trap) from each tree. Above each trap, colored bars represent the average captures of the two lines of trap trays for each distance, and the same distances have the same color. Vertical bars indicate standard error (SE). Letters “a” to “d” show statistically significant differences. For each distance, values followed by the same letter are not significantly different from each other according to Fisher’s LSD multiple range test (*p* ≤ 0.05).

We captured 28,801 mealybug males from the 112 trap trays collected (56 traps per line). The number of mealybugs showed a decreasing gradient from the tree vicinity towards the center of the row (Figure 6). The average of captures in the two trap trays closer to each of the tree trunks (within 1.25 m in diameter) was 55.1 ± 6.1 insects per day, while in those placed at larger distances, the average was 18.3 ± 2.9. There were statistical differences between both groups (F_1,110_ = 29.64; *p* < 0.0001). The higher numbers of insects in the traps located next to the tree trunks show that this is the preferred area for mealybugs to overwinter.

## 4. Discussion

Considering the small size of the trap trays randomly placed on the ground surface and the surprisingly large amount of flying mealybug males captured every year while emerging from the soil, we can infer how big the population of mealybugs overwintering belowground is. Additionally, we should consider that the sex ratio for *P. citri* is about 1:1 [52] and that the estimated amount of males that can emerge during spring within 1 m in diameter around a persimmon tree trunk is higher than 12,000 insects per square meter.

Previous field works evaluating the mealybug life cycle in temperate climates wrongly assumed that the tree trunk or some other areas within the tree canopy were the main overwintering sites and where adult insects diapause (see [12,13,14,18]). However, the results of our novel, specific sampling method give a clear insight into the importance of the role played by the rhizosphere in the life cycle of these insects. In fact, our results suggest that these insects spend about half of the year belowground. If we compare the captures of pheromone traps placed on the same tree but in the plant canopy during the spring and beginning of the summer, we can observe that the average number of insects captured per day is even higher in the ground traps (more than twice as much most days). Taking into account that the action radius estimated in the field for this pheromone is about 150 m [53] and that part of the insects captured in the tree canopy most likely come from the belowground emerging population, we can conclude that most mealybugs spend the winter in the rhizosphere. Moreover, the results of our third experiment indicate that overwintering mealybugs seem to prefer the ground area near the trunk to feed and reproduce. A large majority of mealybugs appear to choose the roots closer to the tree trunk; nevertheless, even at 2 m away from the tree trunk, the maximum distance between tree rows, more than 18 mealybugs per day were captured. This means that virtually all the field orchard rhizosphere area contains overwintering mealybugs.

Captures of aerial pheromone traps showed two peaks, the main one in September and a smaller one in October. Other authors found similar peaks of captures in the Valencia region (see [41]). However, captures of the trap trays were almost zero during these periods, which means that insects in the canopy traps during the end of the summer and autumn originate from new generations of females that developed in the aerial plant zone. The highest female mealybug populations on the fruits were observed in October and November. The gap between the peaks of capture for males and females has been previously described by other authors [54]. One possible reason is that female mealybugs begin to release sex pheromones and are ready to mate the first day after molting, but the duration of the pre-oviposition period takes several weeks [55,56].

Some mealybug species, such as *P. citri*, were thought to have facultatively parthenogenesis [57]. That could partially explain the high numbers of males emerging during the spring of the second year below the trays. However, the most recent intensive studies have demonstrated that all female mealybug species studied, *P. citri* included, produced no viable offspring from unmated females [58,59]. Since no adult males have been observed to look for females at the ground level, the only possible explanation is that gravid females arrive at the rhizosphere during the autumn either by walking from the trunk, being transported by ants [49] or by falling from the tree canopy (with or without fruits). However, we carefully checked that there were no ants, or ant nests, below or around the sampling trays, which suggests that this is not the main way.

Much is now known about the ways in which insects cope with, and respond to, low temperatures. Studies concerning insect overwintering responses are mostly based on areas where subzero temperatures are common (e.g., [60,61]); however, overwintering in temperate climates has been less studied. In the case of the mealybugs, the overwintering strategy of a particular group of insects that can feed, complete their life cycle and reproduce both in the tree canopy area during warmer seasons of the year and in the soil during the winter has not been previously recorded. In fact, one of the first assumptions of researchers focusing on winter ecology, i.e., “overwintering insects cannot feed” (see [61]), is not true for these insects. Feeding on the roots during the winter is a strategy for mealybugs to maintain their energy reserves at a time at which they minimize their cold exposure. This drastic change in habitat and feeding source during half of the year allows the mealybugs to continue with the reproduction process (i.e., complete at least one generation) under the “new belowground conditions” is, to our knowledge, a pattern that has not previously been reported for any other group of insects. Or in other words, this adaptive strategy allows an aboveground insect herbivore to turn into a belowground herbivore during the winter. In fact, none of the main groups for which insect winter ecology has traditionally classified the known overwintering strategies fits with the mealybug’s winter behavior. Therefore, the discovery of this winter survival strategy converts mealybugs, or at least some mealybug species inhabiting temperate zones, into a potential model for a new ecological group with different overwintering behavior than any other described group showing cold avoidance behavior. In order to better understand the winter biology of mealybugs and the characteristics of their below-ground habitat, future studies should include the monitoring of ground temperature during the winter at the root level. This could also help to estimate the moment of spring male emergence. The increasing temperature at 10 cm and 40 cm below the soil surface compared to air temperature and the effect on some overwintering insects has been previously demonstrated (see [62]).

In addition to the implications at the winter ecological level, the results of this research have implications at the agronomical level since, in the many crops where mealybugs become a serious pest, control treatments are based on the tree’s canopy; however, treatments based at ground level should be considered essential. Taking into account that the first seasonal generation of males comes out from the ground during the spring, late winter soil treatments using natural insecticides and/or biofumigation could be a solution to drastically reduce mealybug populations in the crop. For instance the neem (*Azadirachta indica*) oil, which can be applied as a soil drench, has been described to have insecticide action against larval stages of Hemiptera insects due to inhibition of development, suppression of the insects’ appetite and ecdysis defects. At the same time, this biopesticide has a systemic and translaminar activity which means it is absorbed through the roots and acts as a systemic insecticide [63]; thus, this could be a good candidate for soil treatments around the trunk where most mealybugs overwinter. Biofumigation using brassica cover crops and incorporating them into the soil is a promising crop protection strategy that can be used to reduce soil-inhabiting insect pests and pathogens in fruit crops ([64,65]). Besides, the combination of these and other cover crops with animal manures and compost is a method to supply soil mealybug predators with alternative food and refuge. Overall, all practices that promote soil health constitute one of the fundamental pillars of ecological pest management.

## Data Availability

The data presented in this study are available in the manuscript.
